# Evaluation of different robotic grippers for simultaneous multi-object grasping

**DOI:** 10.3389/frobt.2024.1351932

**Published:** 2024-11-07

**Authors:** Werner Friedl

**Affiliations:** Institute of Robotics and Mechatronics, German Aerospace Center (DLR), Wessling, Germany

**Keywords:** robotic hand, hand design, multi-object grasping, robotic grasping, variable impedance

## Abstract

For certain tasks in logistics, especially bin picking and packing, humans resort to a strategy of grasping multiple objects simultaneously, thus reducing picking and transport time. In contrast, robotic systems mainly grasp only one object per picking action, which leads to inefficiencies that could be solved with a smarter gripping hardware and strategies. Development of new manipulators, robotic hands, hybrid or specialized grippers, can already consider such challenges for multi-object grasping in the design stages. This paper introduces different hardware solutions and tests possible grasp strategies for the simultaneous grasping of multiple objects (SGMO). The four hardware solutions presented here are: an under-actuated Constriction Gripper, Linear Scoop Gripper suitable for deform-able object grasping, Hybrid Compliant Gripper equipped with mini vacuum gripper on each fingertip, and a Two-finger Palm Hand with fingers optimized by simulation in pybullet for maximum in-hand manipulation workspace. Most of these hardware solutions are based on the DLR CLASH end-effector and have variable stiffness actuation, high impact robustness, small contact forces, and low-cost design. For the comparison of the capability to simultaneously grasp multiple objects and the capability to grasp a single delicate object in a cluttered environment, the manipulators are tested with four different objects in an extra designed benchmark. The results serve as guideline for future commercial applications of these strategies.

## 1 Introduction

Robotic grasping has received a significant impulse thanks to artificial intelligence, especially in computer vision (Höfer et al., 2021; Durner et al., 2021) and grasp planning (Ichnowski et al., 2020; Mahler and Goldberg, 2017). In particular, for bin-picking, various solutions are now available as commercial products as well (UniversalRobots, 2023; Ocado 2023). These bin-picking solutions work well for packaged goods or grasping one object at a time. These approaches can only increase the number of picks by optimising the robot’s speed, which often leads to grasping errors and, in turn, reduces the number of successful picks. Another alternative to increase the number of picks is grasping multiple objects simultaneously (Harada and Kaneko, 1998).

For grasping objects in a plane using appropriate pre-grasp actions like push or squeeze using a traditional two-fingered gripper, Sakamoto et al. (2021) proposed a strategy for grasping two objects, while Agboh et al. (2022) proposed a strategy for grasping multiple objects simultaneously. In both cases, a significant increase in the number of picks was demonstrated compared to a single object grasp strategy. Yao and Billard (2023) proposed a multi-object grasp synthesis algorithm for a multi-fingered robotic hand, exploiting the kinematic redundancy and pairwise contacts on arbitrary contact surfaces. However, if such a generated grasp plan is actually feasible to be executed on a robotic platform in a realistic setting was not evaluated and tests were conducted on a upright mounted standalone hand, where a human places the objects between the fingers as per the generated grasp plan. Practical challenges such as, grasping a new object without disturbing the already grasped object in hand, the ability of the hand to grasp small objects, or the ability to simultaneously control all the fingers to perform a coordinate multi-grasp need to be addressed in parallel. Chen et al. (2021) used tactile sensing to estimate the number of objects that will remain stably within the hand after a caging grasp. In all cases, the performance is still low compared to the human hand, which is very well suited for grasping multiple objects in terms of its dexterity, passive compliance, soft fingertip or palm, and sensing capabilities. Sun et al. (2022) provides a taxonomy on multi-object grasping with human hand.

Robotic hands designs were also proposed specifically for multi-object grasping (Nguyen and Chow, 2023; Mucchiani and Yim, 2020; Gyeongji and Kahye, 2023). These solutions simplify multi-object grasping with its embedded mechanical intelligence, but are not very suitable for other manipulation actions or single-object grasping. A multi-purpose robotic hand is crucial to solve at-least both multi-object and single object grasping to cater to a wide variety of grasping use-cases like bin-picking, object sorting, household assistance robotics, and agriculture robotics. Jain et al. (2023) presented a solution suitable for both problems, but is not a fully integrated system, due to the valves, pump and sensors are not integrated in the gripper.

**FIGURE 1:**
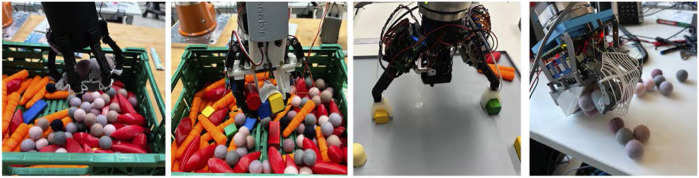
Multi-object grasping with different end-effectors. From left to right: Constriction Gripper, Linear Scoop Gripper, Hybrid Compliant Gripper, and Two Finger Palm Hand.

If we look on food handling, this review of robotic food handling Wang et al. (2022) suggests that there may be a need for specialised, cost-effective manipulators. It would be beneficial to consider that multiple object grasping could potentially reduce system costs by increasing picking rates, which is not really addressed in the review. Additionally, grasping from the bottom could be a useful approach for improving the handling of special objects like Daifuku, which remains an open challenge (Qiu et al., 2023). The demonstrated gripper design Bo et al. (2024) has the capability to grasp multiple foot objects and objects grasped from the bottom, however, there may be opportunities to enhance its target grasping performance due to its scoop size. This paper presents four different end-effectors developed at DLR for multi-object grasping (Section 2): Constriction Gripper (CG), a Linear Scoop Gripper (LSG), Hybrid Compliant Gripper (HCG) equipped with mini vacuum gripper fingertip, and a Two-finger Palm Hand (TPH) with inter-finger net support. HCG and TPH are based on the mechatronics developed for the CLASH hand Friedl and Roa (2020) and has variable stiffness. CG uses the same electronics as the CLASH hand but has no variable stiffness. LSG is actuated by ${\text{qbmove Advanced}}^{R○}$ variable stiffness actuator. All the end-effectors are self-contained and do not need external components such as pressured air or valves for its functioning. They can be mounted easily on any industrial cobot, Figure 1 shows an overview of these end-effectors mounted on a Kuka LWR 4+ robot arm. In Section 3, experimental evaluation of the four proposed end-effectors for single object and multi-object grasping is conducted. The following criteria will be used to evaluate the grasping of a single object: space needed to grasp the object, minimum grasp force, placement accuracy for each object, when releasing. The Linear Scoop Gripper was design specially for soft objects like fish, so the other three gripper are also tested on grasping fish. For multi-object grasping, the maximum number of objects that can be grasped simultaneously, different applicable grasp strategies, and ability to drop or release objects precisely are considered for evaluation.To test both cases a simple benchmark is introduced with following steps:

• Grasp single object between six obstacles in a circle around the object, in each trial the distance between the objects and obstacles is increased, until the grasps are successful

• Place grasped single object on a paper with distance marks, use position control of robot, take picture from below or above.

• Start to grasp simultaneously two objects for ten times, report failures.

• Repeat the prior step by incrementing the number of objects, until failure rate is over 50 percent, report accuracy.

• Place grasped objects on a paper with distance marks, use position control of robot, report accuracy by picture

The requirements of the benchmark are inspired by the food handling use case to handle fruit and vegetable. Necessary materials as the mark paper, the benchmark sheets, the model of the grasp plate and the objects are given in the support material to reproduce the benchmark.The following requirements have to be fulfilled by the grippers:

• Minimum grasp force: <60grams

• single grasp placement error: <5mm

• simultaneously multi grasp placement error: <10mm

• simultaneously multi grasping performance: >=3objects

These evaluations provide valuable insights into the capabilities of the end-effectors to perform different grasping strategies. They also provide baseline guidance (Section 4) for future hardware design aspects that need to be considered to successfully perform both single and multi-object grasping.

## 2 Hardware overview

**FIGURE 2:**
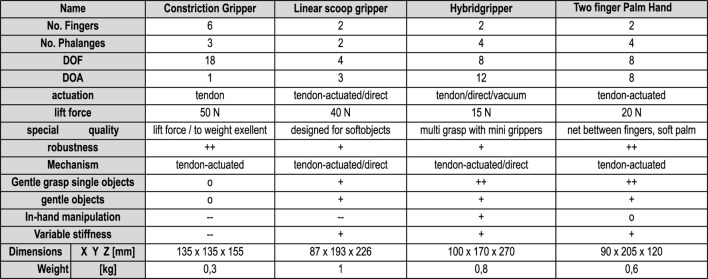
Hardware overview of different end-effectors for multi-object grasping (Explanation for rating symbols: = unsatisfactory; - = fair, o = satisfactory; + = well; ++ = excellent); Explanation: “In-hand manipulation” means, that the gripper can move an object in the hand with dexterity to bring it to a different position or orientation. Explanation dimension: Z ist vertical to the tool flange, X is vertical to the main finger motion (TPH: X to adduction direction).

The different characteristics of the four end-effectors (Figure 1) developed for this experimental study are summarized in Figure 2. They are discussed further in detail in the following sections.

### 2.1 Constriction gripper

**FIGURE 3:**
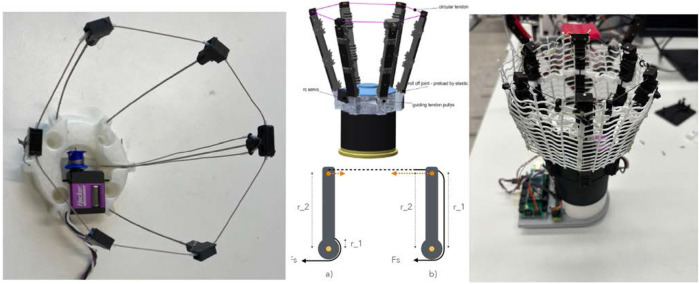
Constriction Gripper. Left: First version of gripper. Middle top: CAD model of updated second version of the gripper used in the experiments. Middle down: A simplified comparison of grip force generation between tendon actuated state of the art gripper a) and constriction gripper b). At a) the grip force is the ratio of $r_{1}$ to $r_{2}$ multiplied by the $F_{s}$ , for b) $r_{1}$ and $r_{2}$ are similar, so $F_{\mathrm{grip}}$ is similar to $F_{s}$ ; Right: CG with installed nets.

The first version of the Constriction Gripper (CG) was designed to be simple, energy efficient, and used for caging spherical objects and net bags (Figure 3, left). Most grippers or hands generate the needed torque to grasp objects in the base of the phalanges, and the required torque on the base motor increases with the length of the fingers. The constriction gripper generates the force in the fingertips with a circular chord that ties the fingertips together, and the tips of the chord are commanded from the base of the gripper. This prototype uses spring steel bars as fingers and a Ditex servo to move the tendon that closes or opens the hand. The tendon was guided in the top of the bars by printed parts with PTFE tube inserts to reduce friction. Initial tests showed a good weight to lift ratio of 40 g tare weight to over 6,000 g of load. For higher loads the bonding of the bars failed.

A second prototype of this gripper (Figure 3, right) was built to better adapt to the object’s form and be more robust against impacts.

The bars were replaced by 3D printed fingers with four phalanges. The joints are roll joints similar to Catalano et al. (2014) and have two elastic tendons to push back the finger. The last phalanges include a pulley for the circular tendon to reduce friction. Five of the six fingers are similar. The sixth finger has only three phalanges to include the routing pulleys to guide the tendon toward the actuator. Initial tests as grasping net bags and spherical fruits as well as pullout tests showed that the push-back force of the return springs in the fingers was not enough to pull the circular tendon back, if the actuator was released. Since the trade-off between good adaptation to the object and good gripper opening is not easy to achieve, an extra release actuator, which can pull back the fingers if needed, was included. The six pull-back tendons are collected on one winder and are loose in regular operation. With this modification, the gripper works well for net bags and spherical objects like apples and mangoes. For the multiple object grasp scenario, elastic structures were included between the second and third phalanges to hold small objects inside the gripper. Furthermore, a net was installed between the fingers. These adaptations allow sufficient contact with multiple objects so that even multi-curved objects like shallots can be securely gripped. Table 1 shows a comparative analysis of both versions of the constriction gripper to give insights of he improvements.

**TABLE 1:** Comparative analysis of both versions of the constriction gripper, Note the inward pull of objects, means that while closing the gripper multiple objects will automatically pulled inside the gripper.

Criteria	Version 1	Version 2
Object adaption	low	excellent
Robustness against collision	low	excellent
stop contact force	high	medium
inward pull of objects	low	excellent
multi grasp performance	low	excellent
weight	40 g	300 g
assembly	simple	more challenging
costs	<100	<250

### 2.2 Linear scoop gripper

**FIGURE 4:**
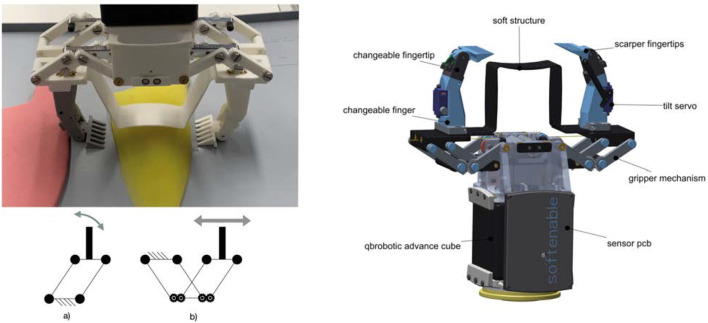
Linear Scoop Gripper. Left top: LSG grasping phantom fish. Left bottom: Schematic of parallel gripper vs. LSG mechanism. Right: Description of different components in LSG. Note that the fingers are easy to change, the grey finger is similar to the white one, build only with a different PLA color. In 2 a newer version of LSG with integrated sensors was used for the photo. For all test the version without sensors was used.

Linear Scoop Gripper (LSG) was developed using the *qbmove Advanced*, a variable stiffness actuator. The finger kinematics and design enable soft fixture-based manipulation of deformable food items like fish, meat, and cheese slices, which is also the primary focus in the project SoftEnable, E. U., 2022. Traditional grippers, like the Soft Claw gripper qbrobotics (2023) from qbrobotics, have small opening width and do not generate parallel force when grasping bigger objects. Other traditional grippers like the robotiq 2F-85, a very common used gripper in research, needs a robot compensation movement for top grasp, that the fingers stay in contact with the surface during closing, which can lead to imprecise sliding of the scoopers under the object, if robot and gripper motion is not proper synchronised. And specially developed fish and meat grippers MarelFish (2020) are only suitable for gripping conveyor belts, where the objects are at a distance from each other. Hence a new mechanism was developed to overcome these drawbacks, which has simultaneous movement of the fingertips in one plane and small dimensions in the closed state. Each side of the gripper consists of two parallelograms placed behind each other, connected via gears. The inner parallelogram is pulled outward by a return spring, and due to the coupling, the second parallelogram follows at the same angle. A tendon on one side is connected to the actuator winder and guided by a pulley to the finger base on the other side. On actuation, the tendon pulls the two fingers in parallel. Each finger is equipped with a fingertip changer introduced in Friedl and Roa (2021), which allows one to easily change fingertips suitable for a specific task. A scrapper fingertip is ideal for caging deformable food items and multiple objects simultaneously as shown in a lot of papers He et al. (2021), Babin and Gosselin (2018). In contrast to these papers the idea of LSG, is to come very flat under the fish without extra arm movements and not to fold the fish by the closing motion. Gafer et al. (2020) shows a similar closing behavior, but cannot secure the objects against gravity or single pick objects in clutter scenes. The scrapper fingertip in LSG has a bi stable spring mechanism in its joint. Two small Bluebird A10 servos can actuate this joint and switch the scrapper between the two positions. The servos are connected to a sensor board based on Raspberry Pico, which can also be used to read different I2C sensors such as time of flight, tactile, or simple analog sensors like force sensing resistors. The variable stiffness actuator is connected over RS485 to the sensor board and mimics the actuator’s native communication protocol. Therefore, the sensor board acts as an interface to control the variable stiffness actuator and the servos, gather sensor data, and store any calibration parameter specific to the gripper. The LSG is also equipped with a soft and compliant palm fixed to the finger base. It can adapt to the object’s shape while providing a mechanism to cage it and increase the contact surface, resulting in a more stable power grasp. The design of the palm was optimised by experiments to achieve a good ratio between increased contact and softness for less influence on grasp force control. Figure 4 shows the LSG grasping a phantom fish fillet using its embedded soft-fixture-based caging mechanism. The scrapper fingertips can slide under the fish, and the counter palm complies with the fish’s surface and ensures a stable grasp. The counter palm also allows to grasp multiple objects, as investigated in Section 3.3. During the project Softenable the gripper is test on fish and meat grasping to improve the initial design. The first simple test is to grasp the object direct from top, the object position is detected by vision and after grasp the object is then placed on a table. The test was performed with silicon phantoms of seabrass filet, salmon filet and a beef steak with different thickness. To do more realistic tests, the silicon phantom were grasped dry (high friction like meat) and oiled with silicon oil (low friction like fish). Each test was run 10 times and the time was recorded. The gripper has in middle a pick rate of 200 per hours and 100 percent success rate. (Note the robot arm is much slower than the industrial solutions and hence the pick rate lower.) As next medium clutter grasping was test, in this case the object can lie direct to the next object, but do not overlap. For this case a different strategy is used, one scoopper stays nearly vertical and the gripper tilts to get the second scooper parallel and in contact to the surface. The vertical scooper is placed between the object, the other in free space. Now the gripper is closing, while keeping both scooper tip on the surface. The object is pushed onto the flat scooper and the other scooper close in the end also. In this grasp case the gripper shows also good performance, but is more depending on accurate object detection. If the second scooper cannot be placed in free space, both scoopers stay nearly vertical and a simultaneous closing of scoopers and and robot movement is needed. The last case with full clutter, was until now only tested with given object positions, due to the vision must be improved to get good results in clutter. The scooppers tilt motion allows to grasp also in high clutter, but more test are needed including vision. Next versions of the gripper will improve the grasping for cases in which the gripper is tilted to the object, to increase the workspace of the arm.

### 2.3 Hybrid compliant gripper

**FIGURE 5:**
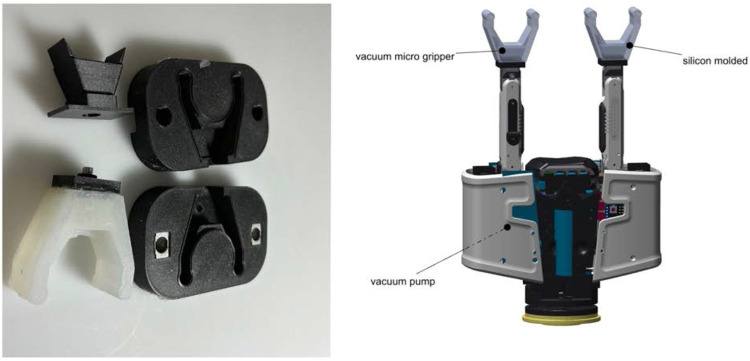
Hybrid Compliant Gripper. Left: Mold for mini vacuum grippper. Right: HCG with mini gripper mounted on the fingertips.

A two-finger gripper was developed based on the thumb models of CLASH hand as a platform for evaluating different tactile sensors in our previous work (Friedl and Roa, 2021). Basically the results out of the paper showed, that following adaptions allow to solve incomplete benchmark for the punnets, but still not perform as fast and simple, if suction cups are used. The fist change was an extra degree of freedom to tilt the modules, which increase the workspace and second are the special designed sensor fingertip, which gives extra information to the planner to found the right grasping points. In logistics areas, a lot of packaging with flat suction able surfaces are used. Hence, the gripper was equipped with a suction cup at the fingertips connected to a vacuum pump, and an electromagnet at the fingertip to grasp ferromagnetic objects, for example cutlery, which led to the creation of the Hybrid Compliant Gripper (HCG). It is possible to simultaneously grasp two objects in different positions with suction at each fingertip as shown in D’Avella et al. (2023). Although it works well for flat objects, for delicate (for instance, berries) or small curved objects (for instance, baby carrots), this approach is not feasible. We transformed the HCG by adding mini vacuum-powered soft grippers at the fingertips to grasp delicate or small curved objects. In contrast to the commercially available mini vacuum grippers like Peab (2023), or Rochu (2023), the custom-built version for HCG can open wider with over pressure and has a different chamber design. The largest object considered in the experiments in Section 3 is a shallot (Figure 7) with a diameter of around 30 mm. The mini gripper is designed for an open grasp span of 35 mm and a minimum closing of 8 mm. An even wider opening should be possible by adding a suitable high-pressure pump. Figure 5 shows the HCG with mini grippers at the fingertip and the molds to produce the mini grippers. The mini gripper is excellent for delicate objects due to low grasp force. By introducing a separate high-pressure line or valves to switch between vacuum and high-pressure, high grasping forces can also be generated. While the HCG with suction cups could grasp two objects simultaneously (D’Avella et al., 2023), the newly adapted version of HCG can grasp two objects with the mini grippers and a third object using the gripper fingers by accommodating it between the two mini grippers.

### 2.4 Two finger palm hand

**FIGURE 6:**
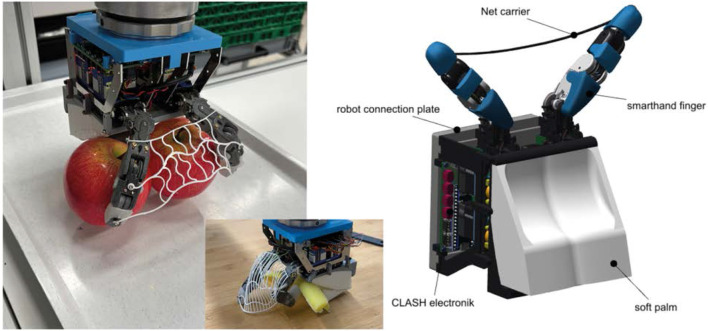
Two Finger Palm Hand (TPH). Left: Grasping two apples between fingers and palm. Middle:TPH with improved net grasping fish phantom. Right: Different components of TPH.

Humans actively use their palm to provide enough support to grasp multiple objects simultaneously (Sun et al., 2022). Inspired by this, a Two-Finger Palm Hand (TPH) was developed (Figure 6). It has a 3D-printed palm and two 3-link fingers closing opposite to the palm. The two fingers, namely index and pinky, are connected by a flexible structure like a net, which provides additional grasping support by constraining the objects and replaces the missing middle and ring fingers. In contrast to the net like string structure in Wang et al. (2021), the net has connections between the single strings, that small objects cannot slide through the single strings. If the fingers build a bag with the net as shown in 13, missing connections would lead to a lot of lost objects. This is due to the fact that the strings are only preloaded by the weight of the object. The initial net was constructed using a single string between the fingers as shown in 6 right to mount nets similar to fish nets. This was then enhanced by a full printed TPE net, shown in 6 (left). The final version can be seen in the middle of 6, which includes a more dense net for multi-object grasping. The mounting interface to the robot arm is in the back of the palm, facilitating palm grasping, which is suitable for multi-object grasping. However, it is not ideal for single object grasps, as the fingers have to grasp the object with fully bent base joints when the last robot joint stays parallel to the object plane. However, since these fingers have a workspace five times higher than a human finger, single objects can be grasped with an adduction grip (Feix et al., 2016), keeping the object lateral between the fingers. This grip usually is not used by humans due to their kinematics. Furthermore, the adduction grip allows the TPH to generate minimal contact torque, as only one tendon in the finger generates the torque, compared to the palmar motion, where three tendons help together to generate the grasping force. The finger design of TPH is also inspired by CLASH hand (Friedl and Roa, 2020). The finger kinematic was optimized by a pybullet simulation using a similar approach as in Roa et al. (2014) to compare different kinematics to get the best inhand-manipulation ability.

## 3 Grasping experiments

**FIGURE 7:**
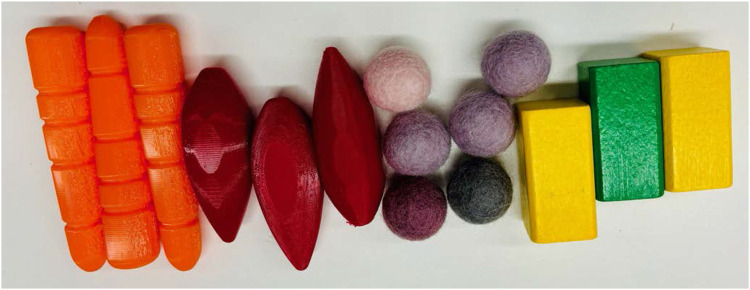
Objects used to evaluate single and multi-object grasping. From left to right: baby carrots, shallots, textile spheres representing cherry tomatoes, and wooden cuboids representing packaged goods.

The Linear Scoop Gripper was design specially for soft objects like fish, so all grippers are tested also on grasping a soft fish-like object. The four end-effectors discussed in the previous section are evaluated for both single-object and multi-object grasping, as both capabilities are crucial. Different evaluation criteria and objects are considered in both cases. A benchmark was designed to compare the grippers in these criteria and the results are shown in the single grasp and multi grasp section. The different-shaped objects (Figure 7) used for evaluation are baby carrots (cylindrical), shallots (ellipsoidal), textile spheres representing cherry tomatoes, and wooden cuboids representing packaged goods. The carrots and shallots are realistic mock ups, 3D printed with thermoplastic polyurethane (TPU) material. Due to the different materials, the friction is also different for all the objects.

### 3.1 Single object grasping

**FIGURE 8:**
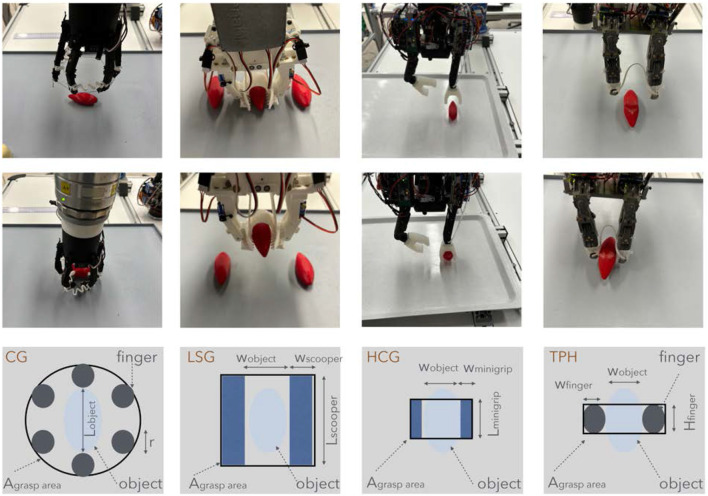
Grasping single items. From left to right: Constriction gripper, SoftEnable linear scoop gripper, HCG with suction fingertips, Two finger Palm Hand; Last row shows the space around each gripper to place it in clutter areas.

Table 2 top summarizes these main performance measurements for single object grasp. Humans grasp delicate objects such as strawberries in general using precision grasp in order to precisely control the grip force. Simultaneously grasping multiple objects brings the risk of damaging the objects. Moreover, single-object grasping is an essential skill for many scenarios and should not be compromised for multi-object grasping. Therefore, the capability of the four proposed end-effectors to grasp a single object was experimentally verified (Figure 8).

**TABLE 2:** Benchmark results for both single and multiple object grasping. (Explanation for rating symbols: = unsatisfactory; - = fair, o = satisfactory; + = well; ++ = excellent. For the placement the rating symbols mean: over 50 mm; - = 30–50 mm; o = 15–30 mm; + = 5–15 mm; 0–5 mm placement error), Note: 30 g minimum grasp force allow to grasp safe strawberries, 50 g is borderline. Note the needed space around the object, is the average for all four tested objects. The multiple grasp success rate is the average for all objects from two up to six objects.

Single grasp	CG	LSG	HCG	TPH
Minimum grasp force (g)	320	50	1	20
Calculated Space around the object $\left(mm^{2}\right)$	2338	2500	638	1200
Versatility in grasping objects	+	o	+	+
Single object placement	o	+	+	+
Benchmark: Space around objects $\left(mm^{2}\right)$	7912	3361	2885	3336

multiple object grasping	CG	LSG	HCG	TPH
Carrot	4	3	3	4
Shallot	6	3	3	6
Textile sphere	8	1	3	9
Wooden cuboid	5	3	3	6
Benchmark:multiple grasp success rate	75	41	45	72
Applicable Strategies	2	1	1	3
Placement Accuracy	o	+	++	-

An important criteria to compare these end-effectors is to consider the space required around the object to grasp it, which corresponds to the specific geometry of the end-effector. This information could be used in a finger position planner as shown in Sundaram et al. (2020). The needed area for the fingertips of the CG can be seen as a hexagon with the fingertips in the edge of it. A motor motion reduces the perimeter radius r of the hexagon by the function (Equation 1):

$$r=\frac{\phi \;R_{pulley}}{6}$$ (1)

where ϕ is the motor rotation angle of the used servo. The starting radius of the gripper is 70mm . Due to the symmetrical motion of the fingers, the Equation 2 for free area around the object is then:

$$A_{\text{grasp area CG}}=\frac{3\;\sqrt{3}}{2}a^{2},\;with\;a=\frac{L_{object}+2\;r}{2}$$ (2)

For example, for a shallot with a length of 50mm , a radius r of 5mm fits, which gives a required area of $2338mm^{2}$ . LSG grasps the shallot with folded fingertips (Figure 8) to prevent the flexible palm from pushing the object out. Hence, the minimum projected area A on the plane is then shown in Equation 3:

$${A=l\;\left(w\;+\;2\;b\right)}_{\text{ with l }=\text{length of scarper fingertip; w }=\text{ width of object and b }=\text{ width of scooper }}$$ (3)

For the same shallot of 50mm length, the result is $2500mm^{2}$ , slightly larger than for the CG, but the LSG requires less safety distance than the highly under actuated CG.

The needed space for the HCG is $22\;mm\;x\;\left(9+width\;of\;object\right)\;mm\;=\;638\;mm^{2}$ for one mini gripper (object width 20), hence it is smaller than for the TPH, which needs $2\;x\;16mm\;x\;\left(19mm+width\;of\;object\right)=\;1216mm^{2}$ . An adaption of the TPH fingertips size is possible by changing them rapidly, as the hand uses a similar interlock system as in HCG.

**FIGURE 9:**
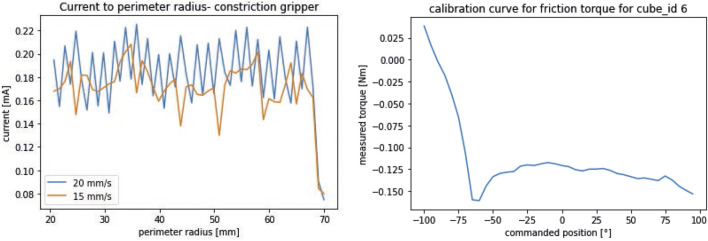
Performance measurements for grippers without direct grasp force measurement. Left: Current consumption of CG depending from the closing velocity for contact measurement; Right: friction torque of LSG. This measurements are needed to control the grasp force of the grippers.

Another important feature for the end effectors is that they can grasp delicate objects gently, as shown for instance for CLASH in Friedl and Roa (2020). The CG has no variable stiffness actuation nor other contact sensors are available, compared to the rest of the grippers. The only sensor which can be used is the current sensor inherited from the CLASH electronics, which is measured by the ZXCT102, a low offset high-side current monitor. The current consumption depends mainly from closing velocity and at a smaller fraction on the closing position. Figure 9 shows the current consumption in a typical setting. These values can then be used for setting a stop condition. With a force gauge calibrated for 30g , ten measurements are performed. As result, the minimum contact force is 320g . To reduce this value, an option would be to program the internal servo parameter to set an internal force limit and a fixed closing speed. However, this was not implemented as it is not a promising alternative to a real force sensor due to the lower performance.

For the Linear Scoop Gripper, the internal torque measurement can be used. The variable stiffness actuation has its own friction torque, which is increased by the finger mechanism and the return springs. Therefore, a measurement is needed to get a position-dependant offset for the stop conditions. The motors are moved over the full motion range and the torque and goal positions are stored. This pair can be then used in the stop conditions. For a closing speed of 6mm/s it can stop at around 60g , for full closing speed of 30mm/s with running stop condition requests, it stops at 150g . Note that the open loop maximum closing speed is 150mm/s .

The HCG can stop with an applied force of about 30g at the fingertips. If the mini vacuum grippers are used, the pressure can be set. These grippers can apply very small forces, the maximum contact weight is 50grams , so they are suitable for handling very delicate objects. The TPH can stop at around 20g for lateral adduction grip. The sensor resolution would allow a bit more, but to use it, a friction observer and an inertia model is needed.

#### 3.1.1 Grasping deformable objects

**FIGURE 10:**
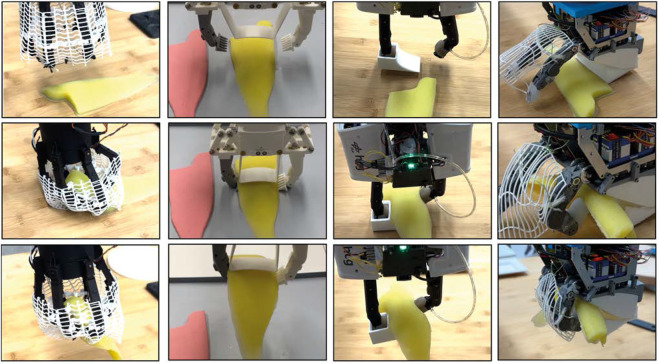
Grasping fish phantom: Left: LSG; Middle; HCG with suctioned slope tool; TPH with human lift strategy.

The grasping of deform-able objects like fish is tested on all four grippers, to get information about the multipurpose capability of the grippers and compare them to LSG, which is special designed for fish and meat grasping. For this test a fish-like silicone phantom has to be grasped from the top. The grasping benchmark for fish and meat described in 2.2 was simplified for this test. The object is placed at a fix position and the pose of the arm to the object is given by human. Also the strategy is given by some inertial first trials for each gripper. The grasping was performed for the fish 10 times and can show only a initial guess, how the gripper performance, for this task, due to the focus of the paper is multiple object grasping. The CG fail due to the opening of the finger is not enough to cage the phantom. Landing with some fingers on the object and closing works, but is very rough to the fish, and is not considered as successful due to marks on the fish. The failing of CG can be explained by its finger form which not allow sliding under the fish as HCG AND LSG can do. Also lifting the fish partly with one finger and then placing the next under the fish as the human or the TPH can do, is not possible with only one degree of freedom. The HCG needs to change the fingertips to grasp the fish, if the mini vacuum gripper are not optimizing for this new task by including nail like structures. With scoopers like on the LSG it is not a big problem due to the four degrees of freedom in each finger. More interesting for a multipurpose approach is, if by suction a tool can be taken, to allow to slide under the fish. This works well as long the fish gets not to heavy to push away the tool, while lifting it. For the TPH are more like human strategy is taken as show in Figure 10. One finger push the fish to palm, the slide on the table to get under the fish and lift it then up, the second can support the grasp.

Out of the experiment two successful fish grasping strategies can be observed, Fingertip with a flat low friction design like scoopers can slide under the fish to grasp it. The needed force to bring the deformable object on the scoopers, can be taken out of the Equation 4 of the inclined plane.

$$F_{push}=\mu \;mg\cos\left(\alpha \right)\;+mg\sin\left(\alpha \right)$$ (4)

A low angle α for the scoopers form decrease the needed force, but also increase the structural problem of the scoopers. Also leads a low α to a knife like form, which can cut the object, if the scoopers not sliding proper on the surface. If the first solution is not possible due to the finger form and friction behavior at the finger tips or rough grasping surfaces, the partial lift strategy as shown at TPH is the right one, but needs more degrees of freedom. Minimum six degree of freedom would be needed for grasping, hence a gripper with two parallel fingers with each two active degrees of freedom is the simplest solution, if a passive coupling between the distal and the middle link is given. The opposing grasp area, could be the palm as at TPH or a passive linear compliant finger, which mimics the thumb.

### 3.2 Benchmark for single and multi object grasping

**FIGURE 11:**
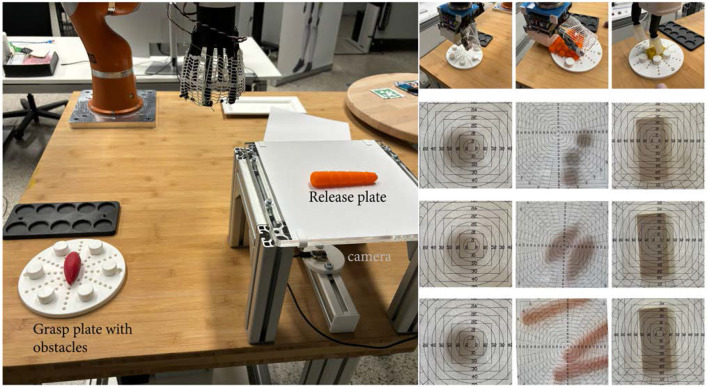
Benchmark setup to test single and multi object grasp; right: grasp trials and placing for different grippers. TPH is able to grasp a high number of objects, but the placing should be improved by better opening motion.

The setup for the benchmark consists (see Figure 11) of a grasp plate with adaptable clutter, this clutter can be changed by six obstacle objects. Furthermore it consists of a release plate to measure the placement. On the release plate is a human readable scale on paper which gives the accuracy of the placement. For simpler and fast evaluation a mobile phone with a face camera or a USB camera can be mounted under the transparent release plate to take pictures of the placement. The position for the grasp and release is one time taught for each gripper and then the grippers has to grasp each object 10 times with obstacles near as possible to the grasp object and release them on the plate. Afterward the obstacles are putting one slot out to and the test is repeated. If the test runs successful for over 100 percent in the grasp phase, the maximum clutter density is found for the gripper. The position of the obstacles has to be report in the benchmark sheet, beginning with clockwise at 12, for this the plate should be mounted on the table, that the two mounting holes are parallel to the *y*-axis of the robot world frame. The mounting hole should be count from the middle and then report in the benchmark documentation sheet. The average of the six position is than taken to calculate the minimum space around the center of the object to grasp it. This is clear a approximation and could be improved by more complicated model than a circle. The Equation 5 to calculate the minimum grasp area is:

$$A={\left(x+o\;10\right)}^{2}\;pi\text{ ; with x }=\text{distance of first hole; o }=\text{ obstacle position}$$ (5)

The plate has two different slots systems, the first aligns with the mounting holes and has a minimum distance for the first hole of 25mm , the second is rotated 30degrees and has a minimum distance of 20mm , The results for the needed space fits quite good to the calculated ones for most tested hardware, for example for the LSG the value out of the benchmark for the shallots is $3670\;mm^{2}$ minus the cross area of the shallot with $1170\;mm^{2}$ fits quite good to 2. TPH and HCG differs a bit more due to the circle area approximation, based on object and finger tips on the benchmark plate instead real area in the calculation. The CG behaves much worse than in calculation, due to the fingers bend during closing mainly in the distal joints, which is good for pushing objects into the net, but on the other side increase the space around the object. The needed space is mostly three times higher than in calculation. The release plate is raised to the grasp plate to allow imprecise placed object to fall off, the results for the placement can taken out the pictures. Objects which are not seen on the pictures are handled as failure. The placement errors for single grasp are mostly less then 5 mm, which is a good result for this intrinsic compliant grippers.

The results of the benchmark are shown in Table 2 and also in detail be include as addition materials.

### 3.3 Multi-object grasping

**FIGURE 12:**
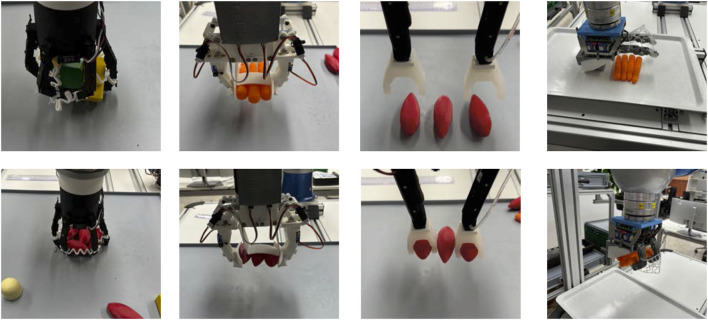
Multi-object grasping performed by different end-effectors. First (from left, top and bottom): CG grasping 5 wooden cuboids, CG grasping 6 shallots. Second (from left, top and bottom): LSG grasping 3 carrots, LSG grasping 3 shallots. Third (from left, top and bottom): HCG in pre-grasp approach to grasp 3 shallots, HCG after grasping 3 shallots. Fourth (from left, top and bottom): TPH in pre-grasp approach to grasp 4 carrots, TPH after grasping 4 carrots.

**FIGURE 13:**
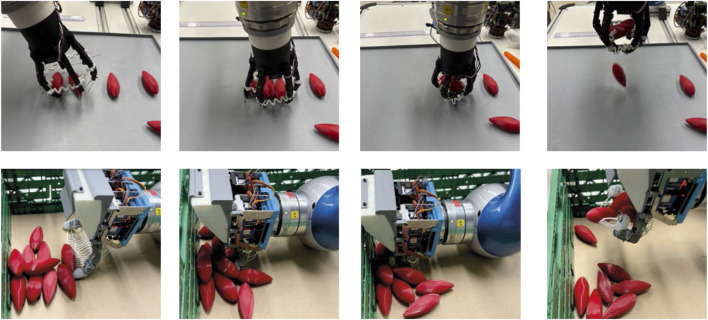
Mulit-object grasping strategies executed by CG and TPH. Top: CG collects objects in a linear motion for a group and grasp strategy. Bottom: TPH groups objects against the bin’s wall for a environmental constrained grasp strategy.

To evaluate the multi-object grasping capability of each gripper, different strategies (top-down grasp, group and grasp, environmentally constrained grasp) were implemented to maximize the number of objects grasped simultaneously. HCG can grasp three objects robustly (Figure 12, third from left) with a top-down grasp strategy. Each vacuum mini gripper on the fingertip can grasp one object and another object can be grasped between the two fingers. But for achieving this, the three objects to be grasped must be placed in a parallel way. Hence, a suitable multi-object grasp planner needs to be developed to search for these object configurations. Due to high opening span of HCG, objects up to 250mm apart can still be grasped. LSG can grasp a maximum of four carrots or three shallows ((Figure 12, second from left)) with a top-down grasp strategy. LSG should be able to grasp more objects with a better suited fingertip design for caging. Although the scrapper fingertip design presented in this work can grasp multiple objects, it is more ideal for thin or deformable food items. CG has the ability to grasp more than three small objects using top-down grasp strategy (Figure 12, first from left), provided they do not fall through the open space between the fingers upon closing. To avoid this, a compliant net joining the adjacent fingers can be used. When the objects are not clustered and far from each other, a group and grasp strategy (Figure 13 top row) can be employed. The gripper is tilted and placed on top of the object such that only three fingers are in contact with the object plane. Using a Cartesian linear motion planner, the arm can move to group more objects for simultaneous grasping. TPH can grasp multiple objects employing all three strategies. First, a top-down grasp (Figure 12, fourth from left) can be used by constraining the objects against the palm while closing the fingers. Up to four carrots can be grasped by TPH from the top. Similar to CG, TPH and also perform a group and grasp strategy. In addition, TPH can grasp multiple objects by constraining them against environmental constraints (Sundaram et al., 2020) such as the walls of the bin (Figure 13, bottom row). The objects can be grouped against the bin’s wall to constrain them and with the help of the net between the fingers, up to six shallots can be grasped simultaneously, this test was performed 5 times and the average was taken. By mounting the hand on a suitable wrist, as presented in Friedl and Roa Garzon 2019, the use of environmental constraints for grasping can be more robustly achieved.

In addition to grasping, the ability of the end-effectors to place the grasped objects precisely is also important for packing or delivering the objects. HCG is the most suited hardware to achieve this due to known object position in the fingers. Precise placement can be achieved by first placing the object between the fingers, followed by the objects in the mini vacuum grippers. LSG is also also robust as all the objects stay parallel within the gripper upon closure. Placement can be achieved by either opening one or both the scrappers, or by opening the fingers. The placement of objects grasped by CG and TPH is relatively inaccurate as they can stay in any orientation within the hand after grasping. For specific shapes, for instance, cylindrical objects like carrot can be placed accurately by TPH. This is due to the inherent shape of TPH that is suitable for constraining specific shapes between the finger and the palm. But if to many carrots are taken, the placement can get even worse, than at the CG as shown in 3.2. For each end-effector, the maximum number of objects that can be grasped, the number of grasp strategies applicable, and the placement accuracy are summarized in Table 2.

## 4 Discussion

All the four end-effectors presented and experimented in the previous sections were successful in grasping single objects, multiple objects, and placing them. If the results are compared with the requirements, the CG had problems to fullfill two of them due to no direct force measurment and only one degree of freedom. LSG and HCG can both fulfil the requirements, the design of LSG is simpler and has some advantages in maximum number of objects, which can be grasped simultaneously. HCG is better suited for grasping simultaneously two delicate objects and placing them. TPH cannot fullfil the simultaneously multi grasp placement error with the simple release, which was programmed, but has similar good simultaneously multi grasping performance as CG paired with very low contact forces and better single grasp performance. However, each end-effector is better suited for different conditions, depending on which task is more critical. The different single and multi-object grasping experiments discussed in the previous section provide valuable insights into the design aspects that need to be considered for the end-effectors for different use cases.

For grasping single object, the end-effectors need varying space around the object for grasp interaction (Table 2). A two-finger end-effector is the most suited one if the objects need to be grasped from clutter. With suitable sensorization, gentle grasping can be achieved as well. For the placing the grippers are all quite accurate, the benchmark could be improve to get more comparable results with 3D printed placement pattern, to compensate human placement errors, which can be seen in 3.2 for example grasping the wooden blocks by the HCG. The vertical position is changing mostly by human placement. Furthermore a second camera looking on the grasp plan, helps to analyze grasp failures better and documents the obstacle positions.

**FIGURE 14:**
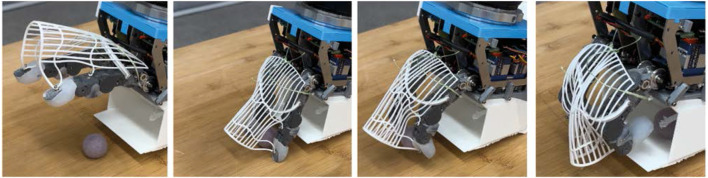
Storage grasp of delicate object. From left to right. The gripper is positioned to grasp the object; Grasping object by adduction grasp; inhand manipulation of object by fingers to place it inside storage; object in storage.

For multi-object grasping, a high contact area to support and stabilize the objects is crucial. This can be done by many fingers as in CG, or by the soft palm in LSG, or with a combination of net and palm as in TPH. So an ideal solution would be a multi-fingered hand, with at-least two highly actuated fingers to enable gentle single object grasping and more than two under-actuated fingers to support the multi-object grasping with more contact area. Adding nets between the fingers can improve the capability to grasp very small objects. Finger kinematics can also be optimized to achieve maximum contact area. A fixed coupling between distal and middle joint allows for a contact only at the tip of the finger. Contact on all phalanges can be achieved by adding one more actuator or a return spring in the distal joint of an under-actuated finger. Furthermore, introducing a stiffness joint brake mechanism similar to the one shown in Friedl et al. (2018) for a variable stiffness finger in TPH can provide a better grasp against the palm, by giving more contact area, between fingers and palm. Considering net between the fingers and improved soft palm surfaces can be transferred to all human like multi-finger hands. For grasping many delicate objects and reducing arm motions, an integrated storage in the gripper could be a solution to reduce transport time and costs. To test this, TPH got a counter part to the palm, to generate a storage. This storage is printed by TPE and as a volume of approximate $83cm^{3}$ . It wraps around the palm structure and it is fixed by two screws at the end of the palm. The flexible material allows for adaptation to different object sizes and could be used as a scoop to push, for example, flat objects into the storage. The single object is grasped by the adduction grasp shown in 3.1 and then by inhand manipulation stored in the palm. The result can be seen in Figure 14 and works only due to the special kinematics of the fingers. For transport the fingers can close the storage and for release the hand is tilted. A storage could also be integrated in HCG to take more small objects, by the small vacuum grippers.

If precise object placement objects is crucial, HCG with the mini vacuum grippers is better suited, but then without a storage. By increasing the number of fingers to more than two, even more objects can be grasped simultaneously. The drawback in this case would be increased system complexity, size, and cost.

## 5 Conclusion

This paper presented four robotic end-effectors for the simultaneous grasping of multiple objects. Furthermore, single-object grasping performance, an essential skill that should not be compromised, was also analyzed. A benchmark is introduced to test the single grasp and placing performance, and furthermore the simultaneous multi grasp performance. The benchmark can help to compare better different designs and improve the results of new developments relative to grippers with force sensing. The simplest solution, the Constriction Gripper, has six fingers with three degrees of freedom each and is actuated by one servo, which pulls in a round rope that runs through the fingertips and thus bends the fingers. This gripper performs well for multi-object grasping, as the fingers can wrap around many objects, and the net between the fingers holds the objects inside the gripper. It is not a suitable solution for single-object grasping in cluttered environments and delicate object grasping due to the large grasp space around the object and no accurate force sensing. Also, the gripper is not precise for placing the objects. To improve single-object grasping, an extra motor to actuate two opposing fingers coupled with moving out fingertips can be developed. These fingers could gently grasp objects with a flexible antagonistic spring like CLASH. The Linear Scoop Gripper can grasp up to three small objects and place them precisely. Single-object grasping is also much better than CG, but it still needs a lot of space around the object. An improvement could be an actuated counter palm to better use the small side of the scoopers. However, the scoopers and soft palm can be transferred to other two-finger grippers to significantly improve the performance of multiple object grasping. Furthermore, these two features are ideal for grasping flat or deformable objects. The Two Finger Palm Hand, designed with two high workspace fingers, has good behavior for single-object grasping and can also grasp several objects by using its palm or net. However, multi-object grasping behavior is not predictable. TPH can grasp three or even six objects, depending on the pull strategy and the contact interaction between the objects. A new palm design could improve the grasp against the palm, and the finger kinematics could be adapted to the task. For instance, a finger with only a longer distal phalanges instead of a fixed coupling between the distal and middle phalanges is better suited. A more exciting option for multi-object grasping is to have more fingers like DLR Awiwi hand Grebenstein et al. (2011) and perhaps some net between the fingers. The concept of nets between fingers can be transformed on all multi-finger hands to improve multiple object grasping. The simplest solution is a glove for a human-like hand with “webs” between each finger. The Hybrid Compliant Gripper HCG with mini-vacuum grippers at the fingertips shows good single-object grasp performance and can also take up to three objects simultaneously. It can also place them precisely, which looks like a very promising solution for delicate and small objects. A new finger kinematic improvement could help grasp objects placed in different rotations and allow new in-hand manipulation. The mini grippers could also be improved to generate higher forces to grasp heavier objects. As a next step, a multi-object grasp planner could be integrated to evaluate realistic bin-picking use cases, for instance, grasping multiple delicate fruits and packaging them. Furthermore analyse of the object stiffness based on haptic contact as shown in Mohammadi et al. (2024) would be an interesting aspect while grasping flexible and delicate objects to get information of the state of the object and the fitting strategy. HCG and TPH should have compareable sensor information, but should allow higher closing velocities due to the variable stiffness actuation.

## Data Availability

The original contributions presented in the study are included in the article/supplementary material, further inquiries can be directed to the corresponding author.
