# Supplementary material for: Evaluation of different robotic grippers for simultaneous multi-object grasping
Source: Front Robot AI. 2024 Nov 7;11:1351932. doi: 10.3389/frobt.2024.1351932 (PMC11578830; doi:10.3389/frobt.2024.1351932)

Multigrasp

	Type		LSG				
**Number of object**		2	3	4	5	6	Success rate
	Ball	100	60	100	0	0	52
	Shallot	100	50	0	0	0	30
	Cube	100	50	0	0	0	30
	Carrot	100	100	50	0	0	50
						Overall	40,5
			CG				
	Ball	100	100	100	100	80	96
	Shallot	100	100	70	50	0	64
	Cube	100	100	100	100	0	80
	Carrot	100	100	100	0	0	60
						Overall	75
			HCG				
	Ball	100	100	100	0	0	60
	Shallot	100	100	0	0	0	40
	Cube	100	100	0	0	0	40
	Carrot	100	100	0	0	0	40
						Overall	45
			TPH				
	Ball	80	80	80	80	70	78
	Shallot	100	80	70	50	0	60
	Cube	100	80	70	60	0	62
	Carrot	100	90	90	90	60	86
						Overall	71,5

single grasp cg

Obstacle	1	2	3	4	5	6	zero distance	Area [mm^2]
**Ball**	4	4	4	4	4	4	15	5674,5
**Shallot**	5	5	5	5	5	5	15	8659,01
**Cube**	5	5	5	5	5	5	15	8659,01
**Carrot**	5	5	5	5	5	5	15	8659,01
**Average**								7912,8825

single grasp LSG

Obstacle slot distance	1	2	3	4	5	6	slot distance [mm]	Area [mm^2]
**Ball**	2	2	2	2	2	2	20	2376
**Shallot**	4	2	2	4	2	2	20	3667
**Cube**	3	2	2	3	2	2	20	2987
**Carrot**	5	2	2	5	2	2	20	4418
**Average**								3.362

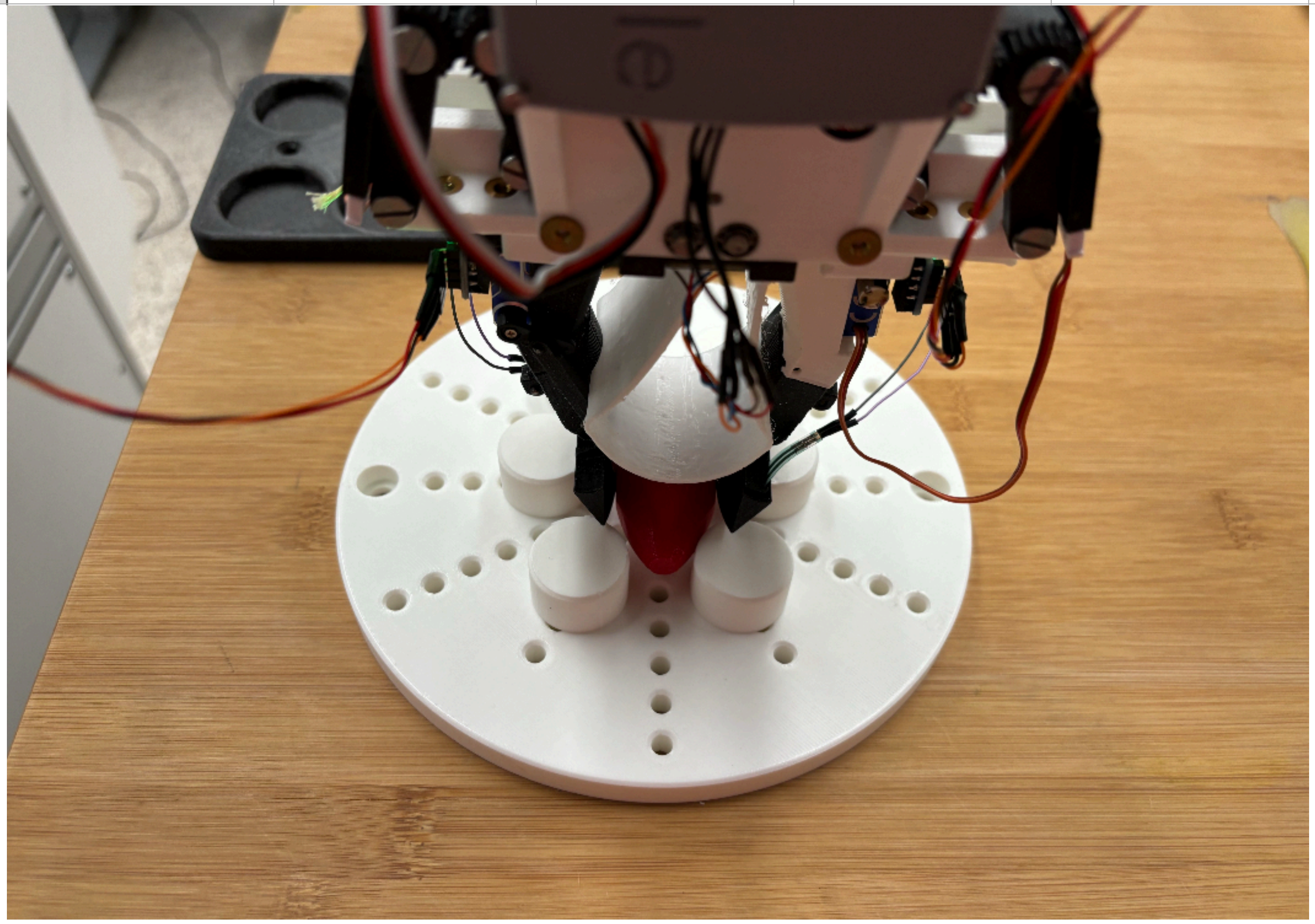

single grasp HCG

Obstacle	1	2	3	4	5	6	zero distance	Area [mm^2]
Ball	2	2	2	2	2	2	15	1590
Shallot	3	3	3	3	3	3	15	3318
Cube	3	2	2	3	2	2	15	2097
Carrot	5	2	2	5	2	2	20	4418
Average								2856

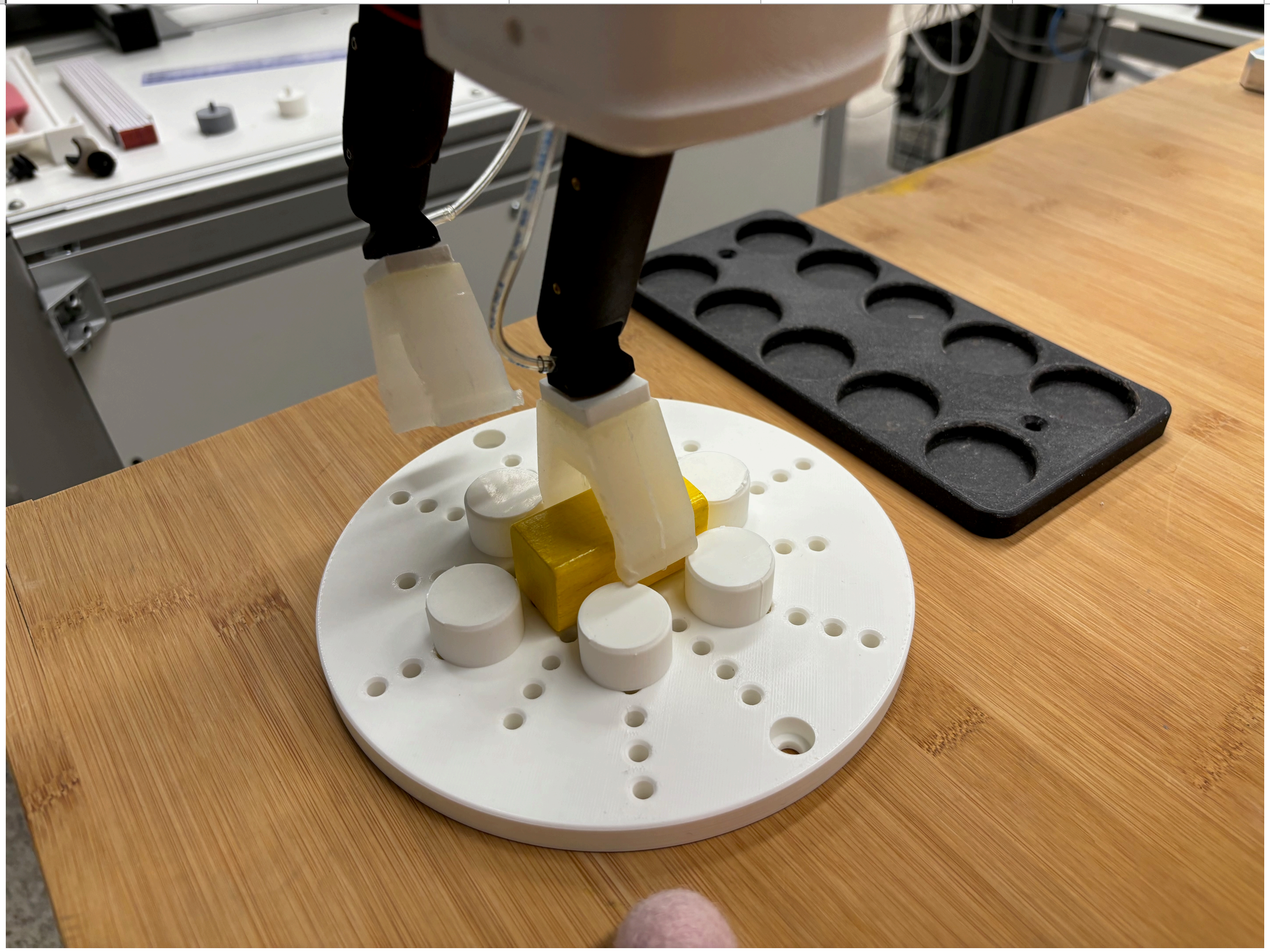

single grasp TPH

Obstacle								
**Ball**	**1**	**2**	**3**	**4**	**5**	**6**	**zero distance**	**Area [mm^2]**
**Shallot**	2	2	2	2	2	2	15	1590
**Cube**	2	4	2	2	4	2	20	3667
**Carrot**	4	2	2	4	2	2	20	3667
	5	2	2	5	2	2	20	4418
**Average**								
								3336

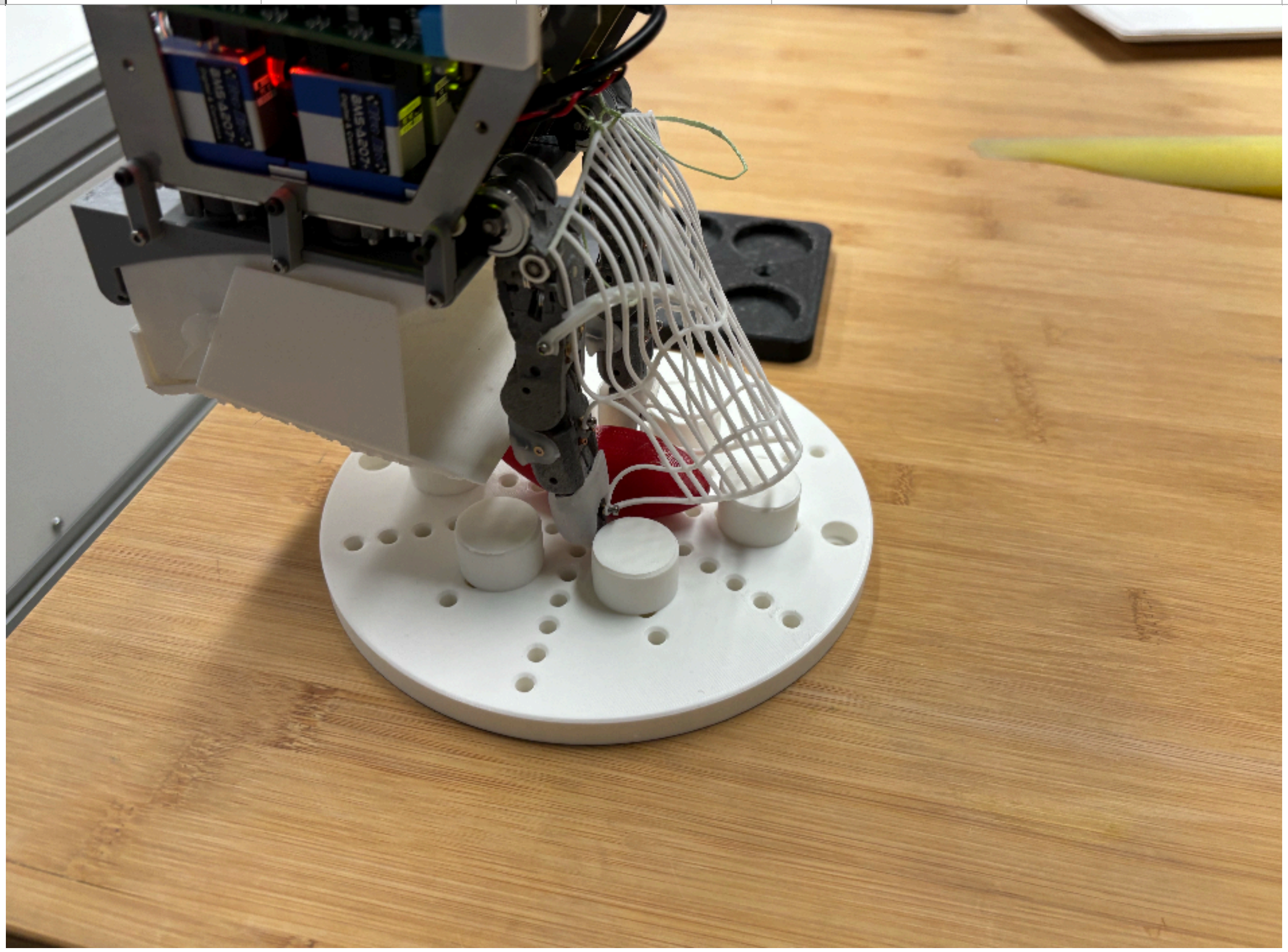

Supplement: Supplementary file 1 [file Table1.pdf]
