# Supplementary figures and images for: Evaluation of different robotic grippers for simultaneous multi-object grasping
Source: Front Robot AI. 2024 Nov 7;11:1351932. doi: 10.3389/frobt.2024.1351932 (PMC11578830; doi:10.3389/frobt.2024.1351932)

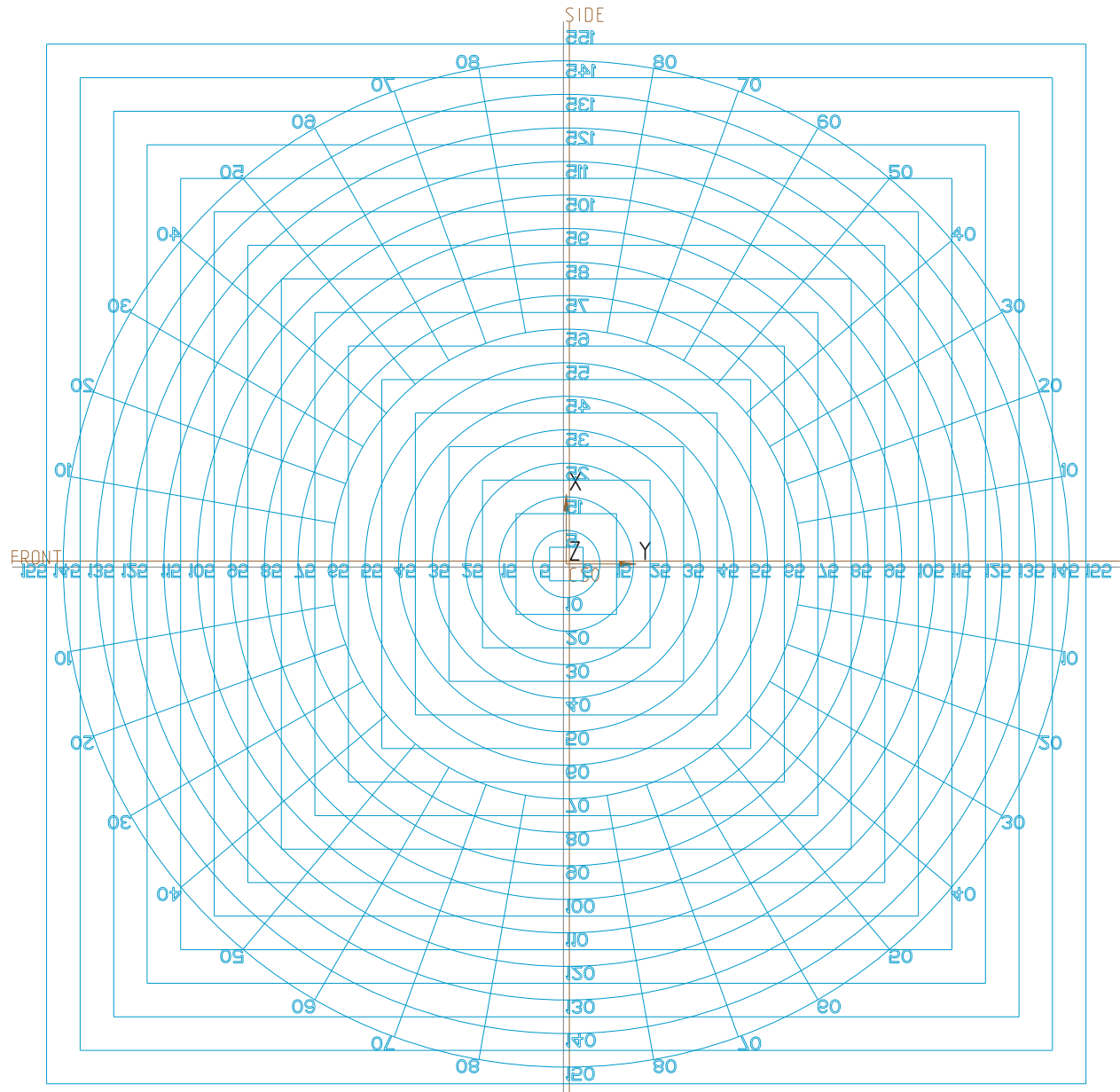

Supplement: Supplementary file 3 [file Image1.pdf]
